# Effects of Diet-Induced Weight Loss on Plasma Markers for Cholesterol Absorption and Synthesis: Secondary Analysis of a Randomized Trial in Abdominally Obese Men

**DOI:** 10.3390/nu14081546

**Published:** 2022-04-08

**Authors:** Sultan Mashnafi, Jogchum Plat, Ronald P. Mensink, Peter J. Joris, Yvo H. A. M. Kusters, Alfons J. H. M. Houben, Coen D. A. Stehouwer, Casper G. Schalkwijk, Sabine Baumgartner

**Affiliations:** 1 Department of Nutrition and Movement Sciences, NUTRIM School of Nutrition and Translational Research in Metabolism, Maastricht University Medical Center, 6200 MD Maastricht, The Netherlands; s.mashnafi@maastrichtuniversity.nl (S.M.); j.plat@maastrichtuniversity.nl (J.P.); r.mensink@maastrichtuniversity.nl (R.P.M.); p.joris@maastrichtuniversity.nl (P.J.J.); 2 Department of Medical Basic Sciences, Faculty of Applied Medical Sciences, AlBaha University, AlBaha 65779-7738, Saudi Arabia; 3 Department of Internal Medicine, CARIM School for Cardiovascular Diseases, Maastricht University Medical Center, 6200 MD Maastricht, The Netherlands; ivo.kusters@mumc.nl (Y.H.A.M.K.); b.houben@maastrichtuniversity.nl (A.J.H.M.H.); cda.stehouwer@mumc.nl (C.D.A.S.); c.schalkwijk@maastrichtuniversity.nl (C.G.S.)

**Keywords:** diet-induced weight loss, cholesterol absorption, cholesterol synthesis, non-cholesterol sterols, visceral fat, subcutaneous fat, intrahepatic lipid, cholesterol precursors, plant sterols

## Abstract

Cross-sectional studies have shown that obesity is associated with lower intestinal cholesterol absorption and higher endogenous cholesterol synthesis. These metabolic characteristics have also been observed in patients with type 2 diabetes, metabolic syndrome, steatosis or cholestasis. The number of intervention studies evaluating the effect of weight loss on these metabolic characteristics is, however, limited, while the role of the different fat compartments has not been studied into detail. In a randomized trial, abdominally obese men (N = 54) followed a 6-week very low caloric (VLCD) diet, followed by a 2 week weight-maintenance period. Non-cholesterol sterols were measured at baseline and after 8 weeks, and compared to levels in lean participants (N = 25). After weight loss, total cholesterol (TC)-standardized cholestanol levels increased by 0.18 µmol/mmol (*p* < 0.001), while those of campesterol and lathosterol decreased by 0.25 µmol/mmol (*p* < 0.05) and 0.39 µmol/mmol *(p* < 0.001), respectively. Moreover, after weight loss, TC-standardized lathosterol and cholestanol levels were comparable to those of lean men. Increases in TC-standardized cholestanol after weight loss were significantly associated with changes in waist circumference (*p* < 0.01), weight (*p* < 0.001), BMI (*p* < 0.001) and visceral fat (*p* < 0.01), but not with subcutaneous and intrahepatic lipids. In addition, cross-sectional analysis showed that visceral fat fully mediated the association between BMI and TC-standardized cholestanol levels. Intrahepatic lipid content was a partial mediator for the association between BMI and TC-standardized lathosterol levels. In conclusion, diet-induced weight loss decreased cholesterol synthesis and increased cholesterol absorption. The increase in TC-standardized cholestanol levels was not only related to weight loss, but also to a decrease in visceral fat volume. Whether these metabolic changes ameliorate other metabolic risk factors needs further study.

## 1. Introduction

Obesity and its associated comorbidities are a major health problem worldwide. An increased visceral fat content, a characteristic of people with abdominal obesity, is clinically the most important form of obesity [1]. Abdominal obesity is strongly associated with insulin resistance, dyslipidemia and hypertension [2], which all contribute to an increased cardiovascular disease risk [1,3]. Recently, we have suggested that overweight and obesity are associated with lower intestinal cholesterol absorption and higher endogenous cholesterol synthesis [4]. These metabolic characteristics have also been observed in patients with type 2 diabetes, metabolic syndrome, steatosis or cholestasis [4]. However, these reported cross-sectional associations do not necessarily imply that weight loss will lead to an increase in cholesterol absorption and a decrease in cholesterol synthesis. To assess whether there is a causal association between weight loss with cholesterol absorption and synthesis, well-controlled intervention studies are needed.

To evaluate changes in cholesterol absorption and synthesis in humans, serum non-cholesterol sterols are frequently used as markers [5]. The cholesterol precursors desmosterol and lathosterol reflect endogenous cholesterol synthesis, while the non-cholesterol sterols sitosterol, campesterol and cholestanol reflect fractional intestinal cholesterol absorption [6]. Using these markers, earlier intervention studies in obese individuals with type 2 diabetes [7,8] or metabolic syndrome [9,10,11] have indeed suggested that diet-induced weight loss increases cholesterol absorption and decreases cholesterol synthesis. However, relations with fat distribution or the different fat compartments, which behave metabolically different [12,13,14], were not studied.

So far, studies evaluating the effects of diet-induced weight loss on cholesterol metabolism in apparently healthy individuals with abdominal obesity are limited. In addition, in most studies that did evaluate these effects, a no-weight loss control group was not included [7,8,9,11]. Furthermore, results have not been compared to those of normal-weight volunteers as a reference population in all previous studies. Finally, in some studies, body weight had not reached a new steady state and participants still had a negative energy balance when serum non-cholesterol sterol concentrations were analyzed after weight loss [8]. Therefore, the aim of this study was to examine the effect of a 6-week diet-induced weight-loss program, followed by a 2-week weight stable period, on markers of cholesterol absorption and synthesis in apparently healthy individuals with abdominal obesity. Results before and after the weight loss in the new steady energy balance were compared to those of normal-weight men. In addition, we examined the relations between changes in markers for cholesterol absorption and synthesis with changes in fat distribution and different fat compartments (visceral fat, subcutaneous fat and intrahepatic lipid) to assess whether changes in aforementioned compartments play a role in cholesterol metabolism characteristics after weight loss. Finally, we used cross-sectional mediation analysis to examine the mediating role of each fat compartment on the relationship between body mass index (BMI) as well as markers for cholesterol absorption and synthesis.

## 2. Materials and Methods

### 2.1. Participants and Study Design

Details of this study have been published before [15]. Briefly, Caucasian, apparently healthy male subjects aged between 18–56 years were eligible to participate when they met the following inclusion criteria: stable body weight (±3 kg in the last 3 months); no diabetes; no active cardiovascular diseases; no inflammatory diseases; no use of antihypertensive medication; no drug or alcohol abuse; no use of medication known to affect lipid or glucose metabolism and no participation in another biomedical trial in the previous 30 days. Both normal-weight men and abdominally obese men participated in this study. Normal-weight subjects had a waist circumference below 94 cm, while this was between 102 and 110 cm in the abdominal obese group. Upon inclusion, the abdominally obese men were randomized to the diet-induced weight-loss group or the no-weight-loss control group, as described previously [15]. The participants in the weight-loss group consumed, under strict guidance, a very-low-caloric diet (VLCD; Modifast; Nutrition et Santé Benelux, Breda, The Netherlands) for 4 to 5 weeks. The aim was to achieve a waist circumference below 102 cm, which is the cut-off value used for the diagnosis of metabolic syndrome [16]. Daily caloric intake of the VLCD was 2.1 MJ (500 kcal) and the content of minerals and vitamins met the Dutch dietary guidelines. Participants in the weight-loss group consumed three VLCD formulas, which had to be dissolved in water, on a daily basis. Hereafter, in weeks 5 and 6, participants consumed three meals of a mixed solid caloric-restricted diet providing 4.2 MJ/day (1000 kcal) daily for one to two weeks. Again, the composition of this diet met the Dutch dietary guidelines. In week 7 and 8, weight maintenance was achieved by providing weekly menus which were adjusted to individual energy requirements. Men allocated to the no-weight loss group were asked to maintain their habitual diet, physical activity level and alcohol consumption throughout the entire study duration. A total of 79 men were included; 25 men were a normal weight (waist circumference < 94 cm) and 54 men were abdominally obese (waist circumference 102–110 cm). One man dropped out before randomization and thus, 53 of the abdominally obese men were assigned to the weight-loss group (N = 26) or no-weight-loss control group (N = 27). Written informed consent was obtained from all participants before the start of the study. The study protocol was approved by the Medical Ethical Committee of Maastricht University Medical Center (METC 12-30-40) and registered at ClinicalTrials.gov (NCT01675401).

### 2.2. Anthropometrics, Fat Distribution and Compartments

Information about overall and abdominal obesity was obtained through measurements of weight, body mass index, waist circumference, hip circumference and waist to hip ratio, as previously described [15]. The volume of the visceral fat and subcutaneous fat compartments, as well as the intrahepatic lipid content, was measured by magnetic resonance imaging (MRI) [17].

### 2.3. Blood Sampling

Venous blood samples were drawn after an overnight fast at baseline and in week 8. Heparin vacutainer tubes were centrifuged at 1300× *g* for 15 min at 4 °C to collect plasma samples. Serum tubes were centrifuged at 1300× *g* for 15 min at 21 °C to collect serum samples. Aliquots were stored at −80 °C until analyzed at the end of the study.

### 2.4. Serum Lipid Analysis

Serum total cholesterol (TC) (CHOD-PAP method; Roche Diagnostic, Mannheim, Germany), high-density lipoprotein cholesterol (HDL-C) (CHOD-PAP method; Roche Diagnostic, Mannheim, Germany), and triglyceride (TG) concentrations—corrected for glycerol levels—were analyzed enzymatically (GPO-Tinder; Sigma-Aldrich Corp., St. Louis, MO, USA). Serum low-density lipoprotein cholesterol (LDL-C) concentrations were calculated using the Friedewald equation [18].

### 2.5. Non-Cholesterol Sterol Analysis

Sterols were measured by gas chromatography equipped with a flame ionization detector (GC-FID) (Hewlett Packard 6890 plus), and with a capillary column (DB-XLB 30 m × 0.25 mm i.d. × 0.25 μm; Agilent Technologies, Amstelveen, Netherlands). Extraction of cholesterol and non-cholesterol sterols was performed based on Mackay et al. [19]. Briefly, a 100 μL plasma sample was saponified with 1 ml of 90% ethanolic sodium hydroxide for 1 h at 60 °C. 5α-cholestane and epicoprostanol were used as internal standards. After two rounds of cyclohexane extraction, samples were derivatized with 30 μL of TMS reagent (pyridine, hexamethyldisilazane and trimethylchlorosilane (9:3:1, *v*/*v*/*v*)). Samples were injected into GC-FID; cholesterol and non-cholesterol sterol peaks were integrated (OpenLab CDS ChemStation Edition; Agilent Technologies, Santa Clara, CA, USA) and their concentrations were calculated relative to the internal standard 5α-cholestane. Non-cholesterol sterol concentrations were standardized for cholesterol concentrations, as determined within the same GC run and expressed as μmol/mmol cholesterol.

### 2.6. Statistics Analyses

Data are presented as means ± standard deviations (SD) unless indicated otherwise. Normality of the data was assessed using the Kolmogorov–Smirnov test. The differences at baseline between normal weight and abdominally obese men were compared with an independent *t*-test. A one-way ANCOVA using baseline concentrations as a covariate was used to examine differences in changes between the diet-induced weight loss and no-weight-loss control treatments. An independent *t*-test was also used to compare differences between the normal-weight men and the abdominally obese men after weight loss. Linear regression analysis was used to examine cross-sectional relations between cholesterol absorption or synthesis markers with anthropometric measures at baseline and with changes after weight loss. Cross-sectionally, we examined whether relationships between BMI (independent variable) with cholesterol absorption or synthesis markers (dependent variables) were mediated by visceral fat, subcutaneous fat or intrahepatic lipids (potential mediators). For this, the PROGRESS plug-in for SPSS version 4.0 (A.F. Hayes, Ohio State University, Columbus, OH, USA) was used (model 4). A *p*-value < 0.05 was considered statistically significant. All data were analyzed using SPSS versions 25.0 and 27.0 for Mac (SPSS Inc., Chicago, IL, USA).

## 3. Results

### 3.1. Clinical Characteristics of Study Participants

A full consort flow diagram is shown in Figure S1. Plasma samples of 25 normal-weight and 53 abdominally obese men were used for measurements at baseline. One participant in the weight-loss group was excluded due to study protocol violations, and three participants dropped out for reasons as indicated previously [15]. In the end, 23 men in the diet-induced weight loss group and 26 men in the no-weight-loss group completed the study. The characteristics of all participants at baseline have been described previously [15]. Briefly, as shown in Table 1, the median age was comparable between normal weight and abdominally obese men. As expected, BMI, waist circumference, waist to hip ratio, subcutaneous fat, visceral fat and intrahepatic lipid contents were higher in the abdominally obese men compared to those with normal weight. At baseline, serum LDL-C concentrations and plasma TC-standardized levels of the cholesterol synthesis marker lathosterol were higher in the abdominally obese men compared to the normal-weight men. On the other hand, TC-standardized levels of all three cholesterol-absorption markers, campesterol, sitosterol and cholestanol, were lower in the abdominally obese men (all *p* < 0.05).

### 3.2. Effect of Weight Loss

In the abdominally obese participants allocated to the diet-induced weight-loss group, mean body weight decreased by 10.3 kg (95% CI: −11.4, −9.2 kg; *p* < 0.001), waist circumference by 11.0 cm (−9.9, −12.1 cm; *p* < 0.001), subcutaneous fat by 0.81 L (−0.93, −0.69 L; *p* < 0.001), visceral fat by 0.85 L (−1.0, −0.67 L; *p* < 0.001) and intrahepatic lipid content by −5.80% (−6.58, −5.02%; *p* < 0.001) compared with the no-weight-loss control group.

Serum LDL-C and triglycerides concentrations were significantly reduced (all *p* < 0.001) in abdominally obese men after 8 weeks of diet-induced weight loss as compared with the no-weight loss control treatment group, as shown in Table 1. HDL concentrations did not differ between two treatment groups after 8 weeks. Compared with the normal-weight group, abdominally obese men had comparable values for serum LDL-C and triglycerides and HDL concentrations at the end of the dietary weight loss period.

TC-standardized plasma campesterol levels were significantly reduced after weight loss (−0.25 µmol/mmol cholesterol (95% CI: −0.43, −0.07 µmol/mmol cholesterol; *p* < 0.05)), while TC-standardized sitosterol levels remained unchanged. In contrast to campesterol, TC-standardized plasma cholestanol levels were significantly increased by 0.18 µmol/mmol cholesterol (95% CI: 0.19, 0.25 µmol/mmol cholesterol; *p* < 0.001). After 8 weeks, TC-standardized campesterol and sitosterol levels remained lower in abdominally obese subjects that lost weight as compared to normal-weight subjects (*p* < 0.001 and *p* < 0.05, respectively), while TC-standardized cholestanol levels were comparable between normal-weight and obese participants after attaining weight loss. Diet-induced weight loss significantly reduced TC-standardized lathosterol levels (−0.39 µmol/mmol cholesterol (95% CI: −0.55, −0.24 µmol/mmol cholesterol; *p* < 0.001)). After weight loss, TC-standardized lathosterol levels were comparable between the normal-weight and obese participants.

### 3.3. Associations between Anthropometrics, Fat Distribution and Fat Compartments with Cholesterol Absorption and Synthesis Markers

Cross-sectional analysis including abdominally obese and normal-weight men at baseline showed significant relationships between markers for cholesterol absorption and synthesis with all anthropometric markers, fat distribution and fat compartments (weight, body mass index, waist circumference, hip circumference, waist to hip ratio, visceral fat, subcutaneous fat and intrahepatic lipid content; all *p* < 0.05) (Table S1). The relation between changes in markers for cholesterol absorption and synthesis with changes in these variables is shown in Table 2. Changes in TC-standardized cholestanol levels after diet-induced weight loss were significantly associated with changes in waist circumference (*p* < 0.01), weight (*p* < 0.001), BMI (*p* < 0.001), hip circumference (*p* < 0.05) and visceral fat (*p* < 0.01). Changes in TC-standardized sitosterol levels were only significantly related to changes in body weight (*p* < 0.05). Changes in TC-standardized campesterol and lathosterol levels with changes in anthropometric measures or intrahepatic lipid were not significantly related.

**Figure 1:**
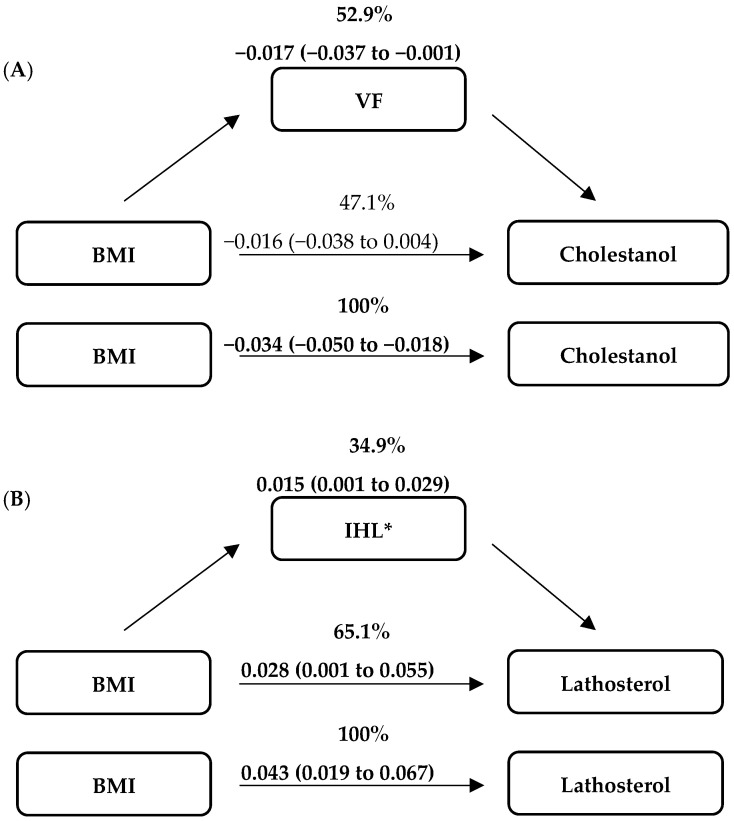
Mediation models of cross-sectional analyses at baseline (*n* = 73) for effects of each mediator on the relationships between BMI (kg/m^2^) and markers of cholesterol absorption (**A**) and synthesis (**B**), expressed in μmol/mmol cholesterol. Data are presented as B (bootstrapped 95% CI). Bold figures indicated for significant effects. VT = visceral fat; IHL = intrahepatic lipid content. * Log-transformed data.

The effect of BMI on TC-standardized cholestanol levels was fully mediated by visceral fat (percentage of mediated effect: −52.9%; bootstrapped 95% CI: −74.0% to −5.5%) and the direct effect of BMI on TC-standardized cholestanol levels was no longer significant (*p* > 0.05) (Figure 1). In addition, the effect of BMI on TC-standardized lathosterol levels was partially mediated by intrahepatic lipid content (34.9%; bootstrapped 95% CI: 10.0% to 44.1%), and BMI had still a significant effect on TC-standardized lathosterol levels (*p* < 0.05). Subcutaneous fat neither fully nor partially mediated the associations between BMI and markers of cholesterol absorption and synthesis.

## 4. Discussion

Diet-induced weight loss reduced levels of TC-standardized campesterol and lathosterol and increased those of TC-standardized cholestanol. After weight loss, TC-standardized lathosterol and cholestanol levels of the (previously) abdominally obese men were comparable to those of normal-weight men. Interestingly, increases in TC-standardized cholestanol levels after weight loss were associated with decreases in waist circumference, BMI, body weight, hip circumference and visceral fat, but not intrahepatic fat and subcutaneous fat volume. Cross-sectionally, visceral fat was a full mediator for the association between BMI and TC-standardized cholestanol levels, while intrahepatic lipid content was a partial mediator for the association between BMI and TC-standardized lathosterol levels.

Our finding of a reduction in endogenous cholesterol synthesis after weight loss (10.3 kg) is in line with earlier studies. A decrease in cholesterol synthesis after weight loss of 6 kg was also observed in a study with apparently healthy obese men, who consumed a hypocaloric diet for 14 weeks followed by a 2 weeks isocaloric diet period [20]. In three studies in obese subjects with metabolic syndrome, cholesterol synthesis also decreased after dietary weight loss of 13 kg, 6 kg and 10 kg, respectively [9,10,11]. Simonen et al. conducted two weight-loss studies in obese, type 2 diabetic patients. Lathosterol levels tended to decrease after a diet-induced weight loss of 15 kg in 3 months [8], and weight loss of 6 kg resulted in a significant decrease in lathosterol levels after a comparable period immediately followed by a weight-stable period up to 2 years [7].

For cholesterol absorption markers, we observed that after weight loss TC-standardized cholestanol levels increased, TC-standardized campesterol levels decreased and TC-standardized sitosterol levels did not change. The question is how these apparent discrepancies for the three different non-cholesterol sterol markers reflecting intestinal cholesterol absorption can be explained. The major diet-derived plant sterols are campesterol and sitosterol [21]. As the diet of the participants in the weight-loss program was different before and after the intervention period, plasma plant sterol levels may also have changed due to different dietary habits and not only due to changes in intestinal cholesterol absorption. Therefore, it can be debated whether TC-standardized plasma campesterol and sitosterol levels truly reflect intestinal cholesterol absorption when major dietary changes are evident. In this particular situation, TC-standardized plasma cholestanol levels may be a better marker for intestinal cholesterol absorption, as cholestanol levels in the diet are very low [22]. We therefore conclude—based on the increase in TC-standardized cholestanol levels—that diet-induced weight loss increased intestinal cholesterol absorption. This conclusion is in line with the study by Simonen et al. [8] that measured cholestanol concentrations after weight loss in type 2 diabetic subjects.

So far, only a few studies have reported effects of diet-induced weight loss on TC-standardized campesterol levels, and findings are inconsistent. In two studies with type 2 diabetic patients, a decrease of about 6 kg induced by 3 months of very-low-energy diet or low-energy diet increased TC-standardized campesterol levels [7], while a trend for a decrease in these levels was found after a reduction of 15 kg induced by a very-low-energy diet virtually free of cholesterol, cholestanol and plant sterols for 3 months [8]. In a third study, weight loss of nearly 10 kg induced by 20 weeks of a free-living diet with a 500 kcal deficiency in daily energy intake, followed by 5 weeks of Mediterranean diet under an isoenergetic, weight-stabilizing period tended to increase total plant sterols levels (campesterol + sitosterol) in obese men with metabolic syndrome compared with a Mediterranean diet in absence of weight reduction [11]. Chan et al. found that campesterol levels decreased in obese men with insulin resistance after consumption of a hypocaloric diet for 16 weeks followed by a 6-week weight-maintaining period [9]. Taken together, studies on campesterol levels after diet-induced weight loss are conflicting. As discussed above, changes in TC-standardized campesterol levels may have been confounded by changes in dietary composition, and therefore may not truly reflect changes in intestinal cholesterol absorption. Information about dietary intake of plant sterols was only reported in two studies; one reported the total plant sterols content in the Mediterranean diet was higher than the North American control diet [11], while the other study used a diet formula free of cholesterol, cholestanol and plant sterols [8]. The total plant sterol level tended to increase in the former study, while a trend of decreased campesterol and sitosterol levels was demonstrated in the latter study. These observations suggest that circulating sitosterol and campesterol concentrations reflect dietary intake and—in contrast to cholestanol levels—are not valid markers for intestinal cholesterol absorption during weight-loss programs.

To the best of our knowledge, this is the first study in apparently healthy abdominally obese men that examined relationships between changes in cholesterol absorption and synthesis with changes in anthropometric measures, fat distribution as well as the size of different fat compartments after diet-induced weight loss. The relation between changes in TC-standardized cholestanol levels with changes in most anthropometric parameters were consistent, i.e., improvements were seen with increased cholesterol absorption. However, for the different fat compartments, changes in cholestanol were related to changes in visceral fat volume, but not to changes in subcutaneous and intrahepatic lipids. Visceral fat is a metabolically active fat depot and is more strongly associated with CVD risk than subcutaneous fat and IHL [12,23,24]. In addition, the amount of visceral fat is positively associated with cholesterol synthesis in obese subjects [25,26], which has been explained by an increased flux of fatty acids from the visceral fat depot via the portal vein to the liver, thereby stimulating hepatic cholesterol synthesis. However, the current study did not find an association between cholesterol synthesis and intrahepatic fat. In the present study, we demonstrated a positive association between visceral fat and TC-standardized lathosterol levels, but we could not find an association between the changes in visceral fat and cholesterol synthesis. This finding agrees with another controlled dietary intervention study in 26 obese men, in which no association was found between changes in visceral fat and cholesterol synthesis [20].

To examine the associations between BMI and markers of cholesterol absorption and synthesis in more detail, we used mediation analysis to cross-sectionally investigate the impact of several potential mediators (visceral fat, subcutaneous fat or intrahepatic lipid) on the direct association between BMI with cholesterol absorption and synthesis markers. Apparently, visceral fat mediated the link between BMI and cholesterol absorption marker cholestanol, while intrahepatic lipid mediated the link between BMI and cholesterol synthesis marker lathosterol. Due to the altered fatty acid flux from visceral fat to the liver, it can be speculated that there is a link between visceral fat volume with endogenous cholesterol synthesis. However, our findings showed a relation between visceral fat and cholesterol absorption, suggesting that fatty acid fluxes might influence intestinal cholesterol absorption. Although we found significant roles for some fat compartments in the associations between BMI and markers of cholesterol absorption and synthesis in the cross-sectional model, this does not eliminate any other mediators related to determinants or metabolic effects of these fat compartments.

Cholesterol synthesis and absorption clearly show a reciprocal pattern [27,28,29], which was also evident in the current study as intestinal cholesterol absorption increased and endogenous cholesterol synthesis decreased after weight loss. An important question arises as to whether changes in cholesterol absorption and synthesis after weight loss may reduce the risk for metabolic diseases. Circulating concentrations of desmosterol, a surrogate marker for cholesterol synthesis involved in the Bloch pathway, were associated with the development of non-alcoholic steatohepatitis (NASH) [30]. These findings were confirmed by Plat et al., who described increased serum desmosterol and lathosterol concentrations in patients with NASH [31]. Moreover, a plant sterol and stanol intervention in rodents showed a reduction in hepatic inflammation, which could be linked to changes in cholesterol synthesis and absorption [31]. In the current study, decreased cholesterol absorption and increased cholesterol synthesis in apparently healthy obese men (without diabetes or the metabolic syndrome) were reversed after diet-induced weight-loss intervention. Whether this also suggests a lower risk of developing type 2 diabetes and metabolic syndrome after weight loss cannot be deduced from these data, but definitely deserves further attention.

## 5. Conclusions

In summary, a 6-week diet-induced weight-loss period followed by a 2-week weight-stable period increases cholesterol absorption and lowers cholesterol synthesis, and resulted in a normalization of cholesterol metabolism characteristics in abdominally obese men as compared to normal-weight men. Moreover, we also showed that changes in cholestanol levels were related not only to weight loss, but also to a decrease in visceral fat volume. Furthermore, mediation analysis results suggest that visceral fat and intrahepatic content play a role in the relationships between BMI and cholesterol absorption and synthesis. Whether this reflects a possible relation with the amelioration of metabolic risk factors needs further study.

## Figures and Tables

**Table 1:** Anthropometric characteristics and plasma cholesterol and non-cholesterol concentrations of normal weight and abdominally obese men at baseline and after 8 weeks with diet-induced weight loss or no-weight-loss control treatment.

	Normal-Weight Group (*n* = 25)	Weight-Loss Group ^1^ (*n* = 23)		Non-Weight-Loss Group ^1^ (*n* = 26)		
	Baseline ^1,2^	Baseline	After 8 Weeks	Baseline	After 8 Weeks	Treatment Effect ^3^
Age (year)	53.7 (25.0–61.6)	52.4 (46.8–61.7)		52.0 (45.4–61.1)		
Body weight (kg)	74.9 ± 8.3 ^###^	98.2 ± 8.1	88.2 ± 7.6	95.9 ± 8.9	96.4 ± 9.2	−10.3 (−11.4, −9.2) ***
BMI (kg/m^2^)	23.3 ± 1.8 ^###^	30.2 ± 1.5	27.1 ± 1.3	29.9 ± 2.5	30.0 ± 2.5	−3.1 (−3.4, −2.8) ***
Waist circumference (cm)	84.9 ± 6.3 ^###^	106.8 ± 3.4	95.9 ± 4.2	106.2 ± 3.8	106.3 ± 4.2	−11.0 (−12.1,−9.9) ***
Hip circumference (cm)	96.6 ± 4.2	108.1 ± 4.4	102.3 ± 4.0	107.2 ± 5.9	107.2 ± 6.4	−5.8 (−6.5, −5.0) ***
Waist to hip ratio	0.88 ± 0.05	0.99 ± 0.03	0.94 ± 0.04	0.99 ± 0.05	0.99 ± 0.05	−0.05 (−0.06, −0.04) ***
Visceral fat (L) ^4^	0.89 ± 0.42	2.17 ± 0.64	1.44 ± 0.51	2.53 ± 0.75	2.62 ± 0.85	−0.85 (−1.0, −0.67) ***
Subcutaneous fat (L) ^4^	1.45 ± 0.51	3.23 ± 0.64	2.44 ± 0.54	2.92 ± 0.81	2.98 ± 0.81	−0.81 (−0.93, −0.69) ***
Intrahepatic lipid (%) ^4,5^	3.43 (3.14–3.69)	4.21 (3.59–6.53)	3.54 (3.08–4.19)	5.34 (4.33–8.31)	6.31 (4.56–9.45)	−0.18 (−0.25, −0.12) ***
LDL-cholesterol (mmol/L)	2.80 ± 0.71 ^###^	3.67 ± 1.03	3.04 ± 0.88	3.70 ± 0.89	3.48 ± 0.77	−0.51 (−0.76, −0.25) ***
HDL-cholesterol (mmol/L)	1.26 ± 0.27 ^#^	1.14 ± 0.16	1.13 ± 0.21	1.09 ± 0.24	1.11 ± 0.26	−0.02 (−0.11, 0.06)
Triglycerides (mmol/L)	1.01 ± 0.48 ^###^	1.63 ± 0.87	1.19 ± 0.54	1.87 ± 0.77	1.92 ± 0.79	−0.60 (−0.89, −0.30) ***
Total cholesterol (mmol/L) **^Ɨ^**	4.02 ± 0.69 ^###^	4.89 ± 0.99	4.15 ± 0.86	5.03 ± 0.78	4.87 ± 0.67	−0.62 (−0.90, −0.35) ***
Campesterol (µmol/mmol cholesterol)	2.39 ± 1.02 ^##^	1.70 ± 0.56	1.54 ± 0.38 ^##^	1.74 ± 0.64	1.83 ± 0.61	−0.25 (−0.43, −0.07) **
Sitosterol (µmol/mmol cholesterol)	1.55 ± 0.70 ^##^	1.08 ± 0.27	1.06 ± 0.19 ^#^	1.12 ± 0.40	1.13 ± 0.35	−0.03 (−0.12, 0.04)
Cholestanol (µmol/mmol cholesterol)	1.53 ± 0.27 ^###^	1.27 ± 0.21	1.45 ± 0.24	1.27 ± 0.27	1.27 ± 0.27	0.18 (0.19, 0.25) ***
Lathosterol (µmol/mmol cholesterol)	1.13 ± 0.46 ^##^	1.47 ± 0.26	1.19 ± 0.24	1.46 ± 0.39	1.59 ± 0.49	−0.39 (−0.55, −0.24) ***

^1^ Values expressed as means ± SD or medians (25–75 percentiles). ^2^ Values are significantly different from abdominally obese participants (*n* = 49) (independent *t*-test): ^#^ *p* < 0.05, ^##^ *p* < 0.01, ^###^ *p* < 0.001. Significantly different from normal weight group (independent *t*-test). ^3^ Values are differences in changes (95% CIs) between treatment groups obtained from one factor ANCOVA with baseline values as a covariate: ** *p* < 0.05, *** *p* < 0.001. ^4^ Data available from normal weight participants (*n* = 24). ^5^ Log-transformed data. **^Ɨ^** Obtained from GC-FID run.

**Table 2:** Results of linear regression analyses to investigate the relation between changes in cholesterol absorption and synthesis markers with changes in anthropometric measures, fat distribution and IHL after weight loss intervention (*n* = 23).

	Cholesterol Absorption						Cholesterol Synthesis	
	ΔCholestanol		ΔCampesterol		ΔSitosterol		ΔLathosterol	
	B	95% CIs	B	95% CI	B	95% CI	B	95% CI
ΔBW	−0.047	(−0.068, −0.025) ***	0.063	(−0.004, 0.130)	0.030	(0.001, 0.058) *	0.011	(−0.039, 0.060)
ΔBMI	−0.149	(−0.223, −0.074) ***	0.203	(−0.020, 0.427)	0.087	(−0.010, 0.184)	0.044	(−0.119, 0.207)
ΔWaist	−0.036	(−0.069, −0.002) **	0.020	(−0.069, 0.109)	0.009	(−0.029, 0.048)	0.027	(−0.032, 0.086)
ΔHip	−0.043	(−0.085, 0.000) *	0.070	(−0.037, 0.177)	0.029	(−0.018, 0.075)	0.005	(−0.070, 0.081)
ΔWaist:Hip	−1.329	(−5.557, 2.899)	−2.827	(−13.046, 7.391)	−0.999	(−5.437, 3.438)	3.867	(−2.900, 10.634)
ΔVF	−0.246	(−0.422, −0.069) **	0.083	(−0.418, 0.585)	−0.032	(−0.250, 0.185)	0.074	(−0.266, 0.414)
ΔSF	−0.066	(−0.362, 0.229)	−0.194	(−0.906, 0.517)	−0.049	(−0.359, 0.260)	0.101	(−0.383, 0.585)
ΔIHL ^†^	0.252	(−0.252, 0.755)	0.376	(−0.857, 1.609)	0.314	(−0.206, 0.834)	−0.280	(−1.115, 0.555)

ΔBW—changes in body weight; ΔBMI—changes in body mass index; ΔWaist—changes in waist circumference; ΔHip—changes in hip circumference; ΔWaist: Hip—changes in waist to hip ratio; ΔVT—changes in visceral fat; ΔST—changes in subcutaneous fat; ΔIHL—changes in intrahepatic lipid content. Significant relationships: * *p* < 0.05, ** *p* < 0.01, *** *p* < 0.001. ^†^ Log transformed.

## Data Availability

The data presented in this work are fully available without restriction.

## Supplementary Materials

The following supporting information can be downloaded at: https://www.mdpi.com/article/10.3390/nu14081546/s1, Figure S1: Consort flow diagram of the study participation; Table S1: Cross-sectional regression analyses to investigate the relations between cholesterol absorption and synthesis markers with anthropometric measures, fat distribution and IHL at baseline in all participants (*n* = 73).

## References

[1] Despres J.P., Moorjani S., Lupien P.J., Tremblay A., Nadeau A., Bouchard C. (1990). Regional distribution of body fat, plasma lipoproteins, and cardiovascular disease. Arteriosclerosis.

[2] Despres J.P., Lemieux I. (2006). Abdominal obesity and metabolic syndrome. Nature.

[3] Ritchie S.A., Connell J.M. (2007). The link between abdominal obesity, metabolic syndrome and cardiovascular disease. Nutr. Metab. Cardiovasc. Dis..

[4] Mashnafi S., Plat J., Mensink R.P., Baumgartner S. (2019). Non-Cholesterol Sterol Concentrations as Biomarkers for Cholesterol Absorption and Synthesis in Different Metabolic Disorders: A Systematic Review. Nutrients.

[5] Miettinen T.A., Tilvis R.S., Kesaniemi Y.A. (1990). Serum plant sterols and cholesterol precursors reflect cholesterol absorption and synthesis in volunteers of a randomly selected male population. Am. J. Epidemiol..

[6] Miettinen T.A., Gylling H., Nissinen M.J. (2011). The role of serum non-cholesterol sterols as surrogate markers of absolute cholesterol synthesis and absorption. Nutr. Metab. Cardiovasc. Dis..

[7] Simonen P., Gylling H., Howard A.N., Miettinen T.A. (2000). Introducing a new component of the metabolic syndrome: Low cholesterol absorption. Am. J. Clin. Nutr..

[8] Simonen P., Gylling H., Miettinen T.A. (2002). Acute effects of weight reduction on cholesterol metabolism in obese type 2 diabetes. Clin. Chim. Acta.

[9] Chan D.C., Watts G.F., Gan S.K., Ooi E.M., Barrett P.H. (2010). Effect of ezetimibe on hepatic fat, inflammatory markers, and apolipoprotein B-100 kinetics in insulin-resistant obese subjects on a weight loss diet. Diabetes Care.

[10] Chan D.C., Watts G.F., Ng T.W., Yamashita S., Barrett P.H. (2008). Effect of weight loss on markers of triglyceride-rich lipoprotein metabolism in the metabolic syndrome. Eur. J. Clin. Investig..

[11] Richard C., Couture P., Desroches S., Benjannet S., Seidah N.G., Lichtenstein A.H., Lamarche B. (2012). Effect of the Mediterranean diet with and without weight loss on surrogate markers of cholesterol homeostasis in men with the metabolic syndrome. Br. J. Nutr..

[12] Fox C.S., Massaro J.M., Hoffmann U., Pou K.M., Maurovich-Horvat P., Liu C.Y., Vasan R.S., Murabito J.M., Meigs J.B., Cupples L.A. (2007). Abdominal visceral and subcutaneous adipose tissue compartments: Association with metabolic risk factors in the Framingham Heart Study. Circulation.

[13] Kershaw E.E., Flier J.S. (2004). Adipose tissue as an endocrine organ. J. Clin. Endocr. Metab..

[14] Tchernof A., Despres J.P. (2013). Pathophysiology of human visceral obesity: An update. Physiol. Rev..

[15] Joris P.J., Plat J., Kusters Y.H., Houben A.J., Stehouwer C.D., Schalkwijk C.G., Mensink R.P. (2017). Diet-induced weight loss improves not only cardiometabolic risk markers but also markers of vascular function: A randomized controlled trial in abdominally obese men. Am. J. Clin. Nutr..

[16] Grundy S.M., Becker D., Clark L.T., Cooper R.S., Denke M.A., Howard W.J., Hunninghake D.B., Illingworth R., Luepker R.V., McBride P. (2002). Third Report of the National Cholesterol Education Program (NCEP) Expert Panel on Detection, Evaluation, and Treatment of High Blood Cholesterol in Adults (Adult Treatment Panel III) Final Report. Circulation.

[17] Kusters Y.H., Schalkwijk C.G., Houben A.J., Kooi M.E., Lindeboom L., Op't Roodt J., Joris P.J., Plat J., Mensink R.P., Barrett E.J. (2017). Independent tissue contributors to obesity-associated insulin resistance. JCI Insight.

[18] Friedewald W.T., Levy R.I., Fredrickson D.S. (1972). Estimation of the concentration of low-density lipoprotein cholesterol in plasma, without use of the preparative ultracentrifuge. Clin. Chem..

[19] Mackay D.S., Jones P.J., Myrie S.B., Plat J., Lutjohann D. (2014). Methodological considerations for the harmonization of non-cholesterol sterol bio-analysis. J. Chromatogr. B Analyt. Technol. Biomed. Life Sci..

[20] Riches F.M., Watts G.F., Hua J., Stewart G.R., Naoumova R.P., Barrett P.H. (1999). Reduction in visceral adipose tissue is associated with improvement in apolipoprotein B-100 metabolism in obese men. J. Clin. Endocrinol. Metab..

[21] Ostlund R.E. (2002). Phytosterols in human nutrition. Annu. Rev. Nutr..

[22] Miettinen T.A., Tilvis R.S., Kesaniemi Y.A. (1989). Serum cholestanol and plant sterol levels in relation to cholesterol metabolism in middle-aged men. Metabolism.

[23] Despres J.P. (2012). Body fat distribution and risk of cardiovascular disease: An update. Circulation.

[24] Liu J., Fox C.S., Hickson D., Bidulescu A., Carr J.J., Taylor H.A. (2011). Fatty liver, abdominal visceral fat, and cardiometabolic risk factors: The Jackson Heart Study. Arterioscler. Thromb. Vasc. Biol..

[25] Adeli K., Taghibiglou C., Van Iderstine S.C., Lewis G.F. (2001). Mechanisms of hepatic very low-density lipoprotein overproduction in insulin resistance. Trends Cardiovasc. Med..

[26] Tobin K.A., Ulven S.M., Schuster G.U., Steineger H.H., Andresen S.M., Gustafsson J.A., Nebb H.I. (2002). Liver X receptors as insulin-mediating factors in fatty acid and cholesterol biosynthesis. J. Biol. Chem..

[27] Alphonse P.A., Jones P.J. (2016). Revisiting Human Cholesterol Synthesis and Absorption: The Reciprocity Paradigm and its Key Regulators. Lipids.

[28] Quintao E., Grundy S.M., Ahrens E.H. (1971). Effects of dietary cholesterol on the regulation of total body cholesterol in man. J. Lipid Res..

[29] Santosa S., Varady K.A., AbuMweis S., Jones P.J. (2007). Physiological and therapeutic factors affecting cholesterol metabolism: Does a reciprocal relationship between cholesterol absorption and synthesis really exist?. Life Sci..

[30] Simonen M., Mannisto V., Leppanen J., Kaminska D., Karja V., Venesmaa S., Kakela P., Kuusisto J., Gylling H., Laakso M. (2013). Desmosterol in human nonalcoholic steatohepatitis. Hepatology.

[31] Plat J., Hendrikx T., Bieghs V., Jeurissen M.L., Walenbergh S.M., van Gorp P.J., De Smet E., Konings M., Vreugdenhil A.C., Guichot Y.D. (2014). Protective role of plant sterol and stanol esters in liver inflammation: Insights from mice and humans. PLoS ONE.

