# Supplementary material for: Effects of Diet-Induced Weight Loss on Plasma Markers for Cholesterol Absorption and Synthesis: Secondary Analysis of a Randomized Trial in Abdominally Obese Men
Source: Nutrients. 2022 Apr 8;14(8):1546. doi: 10.3390/nu14081546 (PMC9030512; doi:10.3390/nu14081546)
Supplement: Supplementary file 1 [file nutrients-14-01546-s001.zip › nutrients-1634601-supplementary.pdf]

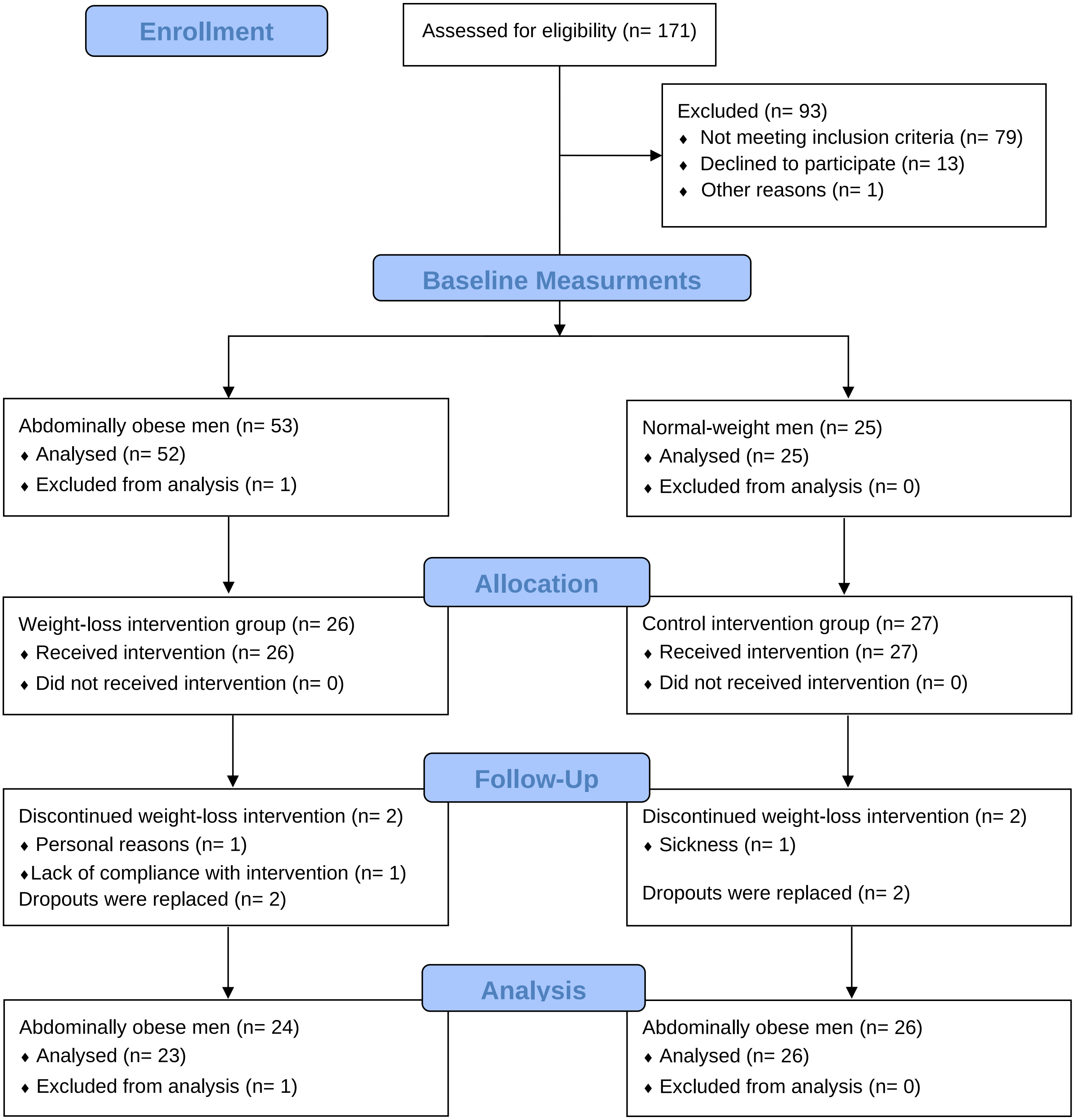

Figure S1. Consort flow diagram of the study participation.

Table S1. Cross-sectional regression analyses to investigate the relations between cholesterol absorption and synthesis markers with anthropometric measures, fat distribution and IHL at baseline in all participants (n = 73).

	Cholesterol absorption									Cholesterol synthesis		
	Cholestanol			Campesterol			Sitosterol			Lathosterol		
	B	SE(B)	P	B	SE(B)	P	B	SE(B)	P	B	SE(B)	P
∆BW (kg)	-0.007	0.002	**0.001**	-0.018	0.007	**0.012**	-0.014	0.004	**0.002**	0.012	0.003	**0.001**
∆BMI (kg/m^2)	-0.032	0.008	**<0.001**	-0.087	0.024	**<0.001**	-0.064	0.015	**<0.001**	0.046	0.012	**<0.001**
∆Waist (cm)	-0.011	0.003	**<0.001**	-0.026	0.008	**0.002**	-0.018	0.005	**0.001**	0.015	0.004	**<0.001**
∆Hip (cm)	-0.009	0.004	**0.047**	-0.025	0.013	0.064	-0.022	0.008	**0.009**	0.019	0.006	**0.004**
∆Waist:Hip	-2.000	0.412	**<0.001**	-4.492	1.311	**0.001**	-2.635	0.862	**0.003**	2.120	0.655	**0.002**
∆VF (L)	-0.121	0.32	**<0.001**	-0.241	0.101	**0.019**	-0.181	0.064	**0.006**	0.116	0.050	**0.023**
∆SF (L)	-0.069	0.031	**0.031**	-0.242	0.092	**0.011**	-0.184	0.059	**0.003**	0.112	0.046	**0.017**
∆IHL* (%)	-0.452	0.152	**0.004**	-1.199	0.458	**0.011**	-0.873	0.293	**0.004**	0.788	0.215	**<0.001**
